# Identification and mapping of stable QTL with main and epistasis effect on rice grain yield under upland drought stress

**DOI:** 10.1186/1471-2156-15-63

**Published:** 2014-05-27

**Authors:** Nitika Sandhu, Anshuman Singh, Shalabh Dixit, Ma Teresa Sta Cruz, Paul Cornelio Maturan, Rajinder Kumar Jain, Arvind Kumar

**Affiliations:** 1 Plant Breeding, Genetics, and Biotechnology Division, International Rice Research Institute, DAPO Box 7777, Metro Manila, Philippines; 2 Department of Molecular Biology and Biotechnology, CCS Haryana Agricultural University, Hisar 125004, India

**Keywords:** Bulk segregant analysis, Drought, Marker-assisted breeding, Rice, Quantitative trait loci

## Abstract

**Background:**

Drought is one of the most important abiotic stresses that cause drastic reduction in rice grain yield (GY) in rainfed environments. The identification and introgression of QTL leading to high GY under drought have been advocated to be the preferred breeding strategy to improve drought tolerance of popular rice varieties. Genetic control of GY under reproductive-stage drought stress (RS) was studied in two BC_1_F_4_ mapping populations derived from crosses of Kali Aus, a drought-tolerant *aus* cultivar, with high-yielding popular varieties MTU1010 and IR64. The aim was to identify QTL for GY under RS that show a large and consistent effect for the trait. Bulk segregant analysis (BSA) was used to identify significant markers putatively linked with high GY under drought.

**Results:**

QTL analysis revealed major-effect GY QTL: *qDTY*_*1.2*_, *qDTY*_*2.2*_ and *qDTY*_*1.3*_, *qDTY*_*2.3*_ (DTY; Drought grain yield) under drought consistently over two seasons in Kali Aus/2*MTU1010 and Kali Aus/2*IR64 populations, respectively. *qDTY*_*1.2*_ and *qDTY*_*2.2*_ explained an additive effect of 288 kg ha^−1^ and 567 kg ha^−1^ in Kali Aus/2*MTU1010, whereas *qDTY*_*1.3*_ and *qDTY*_*2.3*_ explained an additive effect of 198 kg ha^−1^ and 147 kg ha^−1^ in Kali Aus/2*IR64 populations, respectively.

Epistatic interaction was observed for DTF (days to flowering) between regions on chromosome 2 flanked by markers RM154–RM324 and RM263–RM573 and major epistatic QTL for GY showing interaction between genomic locations on chromosome 1 at marker interval RM488–RM315 and chromosome 2 at RM324–RM263 in 2012 DS and 2013 DS RS in Kali Aus/2*IR64 mapping populations.

**Conclusion:**

The QTL, *qDTY*_*1.2*_, *qDTY*_*1.3*_*, qDTY*_*2.2*_, and *qDTY*_*2.3*,_ identified in this study can be used to improve GY of mega varieties MTU1010 and IR64 under different degrees of severity of drought stress through marker-aided backcrossing and provide farmers with improved varieties that effectively combine high yield potential with good yield under drought. The observed epistatic interaction for GY and DTF will contribute to our understanding of the genetic basis of agronomically important traits and enhance predictive ability at an individualized level in agriculture.

## Background

Rice is the staple food of more than 60% of the world’s population and rice consumption is expected to continue to grow as population increases. Globally, rice is grown over an area of about 164 million ha, with an annual production of 723 million tons [1]. More than 90% of the world’s rice is grown and consumed in Asia where 60% of the earth’s population lives [1]. China and India, which account for more than one-third of global population, produce more than half of the world’s rice [2]. The stagnating yield of rice varieties and climate change-related risks are major concerns for world food security. It is estimated that the world needs to produce 40% more rice to feed the population by 2025 [2]. A large portion of this predicted increase has to come from rainfed lowland and upland rice areas, which occupy nearly 38% of the total cropped area and contribute only 21% to total rice production [3].

Drought is the major constraint to rice production in rainfed areas across Asia and sub-Saharan Africa. In the context of current and predicted water scarcity, increasing irrigation is generally not a viable option to alleviate drought problems in rainfed rice-growing systems. Variation in intensity and severity of drought from season to season and from place to place requires cultivation of rice varieties with different levels of drought tolerance in different areas. Rice is highly sensitive to drought stress during reproductive stage, when even moderate stress can result in drastic reduction in grain yield [4,5]. The timing of drought: early season, mid-season, or terminal stage has a major influence on how much yield loss occurs [6]. Therefore, poverty reduction strategies in rainfed areas must focus on stabilizing yields, that is, on breeding varieties with improved yield under drought stress as well as good response to irrigated conditions. To a farmer’s eyes, a drought-resistant cultivar is one that yields better than any other available cultivar, particularly under water-limited conditions [7]. The ability of crop cultivars to perform reasonably well in drought-stressed environments is paramount to ensuring stability of production. The relative yield performance of genotypes in drought-stress and non-stress environments can be used as an indicator to identify drought-resistant varieties in breeding for drought-prone environments.

Upland rice, which represents 12% of the total production area [8], is grown almost exclusively by small-holders for household food security but is prone to damage by drought [9]. Given the high risk of crop loss due to drought, upland rice growers are reluctant to invest in yield-enhancing inputs such as fertilizer, trapping them in a cycle of low productivity [10]. By reducing risk and encouraging farmers to invest in yield-increasing inputs, upland rice cultivars with improved drought resistance could result in greater productivity both in drought years and years with adequate rainfall.

The development of upland rice cultivars with improved drought tolerance is thus an important element in reducing risk, increasing productivity, and alleviating poverty in communities dependent on rainfed rice production. Drought tolerance is considered a complex trait. However, in recent years, considerable progress has been made in the areas of identifying suitable donors and devising effective selection criteria for traits related to drought tolerance [11,12]. These results should hasten the development and delivery of drought-tolerant varieties. Another means to improve breeding efficiency is to identify quantitative trait loci (QTL) with large and consistent effects on yield under drought stress that could be used for marker-assisted breeding (MAB).

Besides the contribution of a single locus, it has been hypothesized that epistasis is one component of the genetic basis of quantitative traits [13]. Epistasis refers to interactions among alleles of different genes, where one gene interferes with the phenotypic expression of another gene [13]. In the last decade, several studies have revealed that digenic interaction is an important component of the genetic basis of rice yield traits [14-17]. Epistasis is important as genetic basis of rice yield not only because there are more epistatic QTL than main-effect QTL but also because a large portion of the main-effect QTL are involved in epistatic interactions [17].

Recently, direct selection for grain yield under drought stress was reported successful at IRRI in improving yield under drought and the feasibility of combining high yield potential with good yield under drought has been demonstrated [11,18]. This breakthrough resulted in the development of several promising breeding lines for the rainfed lowland and rainfed upland [19,20]. Similarly, using molecular markers, large-effect QTL for grain yield under drought stress has been identified [21-27].

Identifying major QTL that show a consistent effect against the backgrounds of different popular varieties occupying a large area in the drought-prone rainfed ecosystem and the use of such QTL to improve drought-susceptible varieties could be an effective marker-assisted breeding strategy. Bulk segregant analysis (BSA) is a cost-effective strategy to identify tightly linked QTL. Recently, some studies have used BSA approach to identify large-effect drought GY QTL [24-27]. BSA involves the bulking of DNA of phenotypic extremes and genotyping of parents along with the bulks with markers polymorphic between the two parents involved in the development of the population. Our study aimed at identifying QTL with a major and consistent effect on GY under reproductive-stage drought stress from a drought tolerant donor, Kali Aus.

## Methods

The study was conducted at the International Rice Research Institute (IRRI), Los Baños, Laguna, Philippines, in 2012 DS and 2013 DS. IRRI is located at 14°13′_N latitude, 121°15′_E longitude, at an altitude of 21 m above mean sea level. The soil type of the experimental field is Maahas clay loam, isohyperthermic mixed Typic Tropudalf.

### Development of mapping populations

BC_1_F_4_ populations derived from crosses Kali Aus/2*IR64 and Kali Aus/2*MTU1010 were used in this study. IR64, a drought-sensitive mega variety widely cultivated in many countries in Asia due to its high-yielding ability, desirable quality traits, and acceptable tolerance for major biotic stresses and MTU1010, a mild drought-tolerance rice variety widely grown in India. The two varieties provide a suitable background for developing a high yielding drought tolerant variety due to the qualities mentioned above. Kali Aus (donor parent) is a drought-tolerant traditional donor from India. It is a medium-duration line characterized by a distinct purple base and tip during its vegetative growth stage. BC_1_F_1_ population was developed by the above mentioned crosses and advanced through selfing and bulking to develop a BC_1_F_4_ population used for the study.

### Phenotyping under reproductive stress (RS) and non-stress (NS) conditions

Reproductive-stage drought stress (RS) and non-stress (NS) trials were established in α lattice design with two replications under upland conditions. Seeds were dry-direct-seeded in the soil using a seeding density of 2 g per linear meter of row, resulting in a seed rate of approximately 325 seeds m^−2^ in 2012 and 342 seeds m^−2^ in 2013. Planting dates were 3 January 2012 and 18 December 2013 for the NS trials and 11 January 2012 and 22 January 2013 for the RS trials for Kali Aus/2*IR64, whereas for Kali Aus/2*MTU1010, planting dates for the NS trials were 3 January 2012 and 20 December 2013 and those for RS were 6 January 2012 and 22 January 2013. Field management of upland trials was done as described by Bernier et al. [21]. In both years, the NS trial was sprinkler-irrigated twice weekly and the RS trials were sprinkler-irrigated twice weekly during establishment and early vegetative growth; irrigation frequency in the latter was reduced at 56 and 40 d after sowing in 2012 and 2013, respectively. Plots were re-irrigated periodically when most lines wilted and exhibited leaf drying. This type of cyclical stress is considered to be efficient in screening for drought resistance in populations consisting of genotypes with a broad range of growth durations [28] and ensures that lines of all durations are stressed during reproductive development. Soil moisture potential was measured using a tensiometer until the crops reached physiological maturity (Additional file 1: Figure S1).

### Data recording and analysis

Days to flowering (DTF) was recorded when 50% of the plants in the plot had exerted their panicles. Plant height (PH) was measured as the mean height of five random plants for each entry measured from the base of the plant to the tip of the panicle during maturity stage. Grain yield (GY) was determined at physiological maturity or when 80–85% of the panicles turned into golden yellow and the panicles at the base were already at the hard dough stage; the harvested grains were threshed, dried to 14% moisture content, and weighed for yield computation (kg ha^−1^).

Data were analyzed using the Statistical Analysis System (SAS v9.1.3) and CROPSTAT. Analysis of variance (ANOVA) was performed for each trait. Correlation analysis was also done in each trial. The means of the lines were estimated using a linear mix model of CROPSTAT, considering replications and blocks within replication as random effects and lines as fixed effect.

The combined analysis over years was calculated as:

$$\begin{array}{ll} \mathrm{Pijkl}= & M+\mathrm{Yi}+\mathrm{Rj}\left(\mathrm{Yi}\right)+\mathrm{Bk}\left[\mathrm{Rj}\left(\mathrm{Yi}\right)\right]+\mathrm{Ll}+\mathrm{LYli}\\ & +\mathrm{eijkl} \end{array}$$

Where, *Pijkl* is a measurement recorded on a plot, *M* is the mean over all plots and both years, and *Y*, *R*, *B*, *L*, and *e* refer to years, replicates, blocks, lines and plot residuals, respectively.

Broad-sense heritability (H) across years was estimated as

$$H=\frac{\sigma_{G}^{2}}{\sigma_{G}^{2}+\left(\sigma_{\mathrm{GY}}^{2}/y\right)+\left[\sigma_{E}^{2}/\left(\mathrm{ry}\right)\right]}$$

where σ _G_^2^ is the genotypic variance, σ _GY_^2^ is the genotype × year variance, σ _E_^2^ is the plot residual variance, and *r* and *y* are the number of replicates and years, respectively.

### Genotyping

Molecular work was carried out at the Molecular Markers Application Laboratory (MMAL) of IRRI’s Plant Breeding, Genetics, and Biotechnology Division. Fresh leaf samples were collected from each entry of a single replication of the NS experiment in both mapping populations at 21 DAS (days after sowing) and underwent dry-freezing using the lyophilizer. The DNA was extracted using the modified CTAB protocol [29]. The agarose gel electrophoresis method was used to check the quality and quantity of DNA. The concentration of the isolated DNA was estimated by comparing band brightness and thickness with a reference λDNA. The DNA samples were diluted with 1x TE into an equal concentration of 25 ng uL^−1^.

Amplification of simple sequence repeat (SSR) markers was carried out as described by Bernier et al. [21] using polymerase chain reaction (PCR). The PCR profile for SSR described by Thompson et al. [30] was used. PCR products were resolved using high-resolution 8% polyacrylamide gel electrophoresis (PAGE) as described by Sambrook et al. [31]. The gel was run in 1x TBE at 95 volts for 1 to 3 h, depending on the product size of the SSR marker. Gels were stained with SYBR Safe^TM^ DNA gel stain and were viewed after 20 min.

### Bulk segregant analysis (BSA), whole-population genotyping, and QTL analysis

A total of 600 rice simple sequence repeat (SSR) markers were tested for polymorphism between the parents, IR64, MTU1010, and Kali Aus. All markers were taken from the published rice genome maps [32] and their physical position (Mb) on the Nipponbare genome was used for an approximate estimation of cM distances by multiplying by a factor of 3.92. For the estimation of genetic distances between markers for QTL mapping, one million bases on a rice chromosome were assumed to be equivalent to approximately 3.92 cM to estimate the genetic distances [32]. These cM positions were used for composite interval mapping (CIM). In our study three hundred BC_1_F_4_ genotypes from each population were used for mapping large-effect QTL for GY, DTF and PH under RS. From each population, 4% of the highest and 4% of the lowest yielding lines were selected based on GY data from the stress trials of 2012 DS and their DNAs were pooled in equal quantities to prepare high and low yielding bulks. For BSA, 134 and 109 polymorphic SSR markers for Kali Aus/2*IR64 and Kali Aus/2*MTU1010, respectively, were used to cover the entire rice genome and to identify markers showing a significant banding pattern for high and low bulks in Kali Aus/2*IR64 and Kali Aus/2*MTU1010 populations, respectively. Markers showing a clear difference in the form of banding patterns coinciding with those of the parents and clearly visible band intensity between the high and low tail bulks were selected. Seven out of the 109 and eight out of 134 polymorphic markers were found to show different banding pattern for low and high bulk tails in BSA in the Kali Aus/2*MTU1010 and Kali Aus/2*IR64 mapping population, respectively and these markers were used to genotype the whole population. Single-marker regression analysis was carried out to identify significant markers associated with GY under RS using Qgene [33]. Additional polymorphic markers on both sides of the significant markers from this analysis were run on the whole population to determine the QTL flanks.

Composite interval mapping (CIM) through QTL Network v2.1 [34,35] was carried out to compute marker intervals, F value and/or probability value, additive effects and broad-sense heritability of significant QTL. Phenotypic variance of the QTL was estimated through composite interval mapping using QGene software [33]. The additive effect as an absolute value varies with differences in severity of drought and does not reflect a proper estimation of the effect in case of very severe drought (low additive effect absolute value) as against mild drought (high additive effect absolute value). To correct this, the additive effect was presented as percentage of the population mean using the formula

$$\mathrm{AE}\%=\frac{\mathrm{AE}\times 100}{\mathrm{PM}}$$

where AE% is the additive effect in percentage, AE is the nominal additive effect and PM is the population mean. AE and PM were calculated using QTL Network.

## Results

### Phenotypic performance of the populations

The mean GY of the population during 2012 DS under stress was 664 kg ha^−1^, whereas GY during 2013 DS was 638 kg ha^−1^, indicating the severity of drought stress in these experiments (Table 1). Kali Aus, the drought tolerant donor, consistently outyielded the recipient parent MTU1010 in the 2012 and 2013 RS trials, except for the NS trial under which MTU1010 showed a 20% yield advantage over Kali Aus (Table 1). A higher yield reduction of MTU1010 was observed under stress conditions as compared with Kali Aus: it showed a 47% and 42% yield advantage over MTU1010 in the 2012 and 2013 stress trials, respectively (Table 1). The best performing line yielded 1654 kg ha^−1^, outyielding the tolerant parent by 47%, while the least performing line yielded only 118 kg ha^−1^ (Table 1). The Kali Aus/2*IR64 mapping population showed an overall mean GY of 722 kg ha^−1^ and 582 kg ha^−1^ during the 2012 DS and 2013 DS stress trials, respectively. As compared with NS yield, 50% GY reduction was incurred during the 2012 DS RS trial, while only 39% GY reduction was observed in the 2013DS RS trial. Under RS condition, Kali Aus consistently outperformed IR64, with a yield advantage of 45% in the 2012 DS. Under RS, GY ranged from 123 kg ha^−1^ to 1890 kg ha^−1^ during 2012 DS and from 156 kg ha^−1^ to 2863 kg ha^−1^ during 2013 DS. On the other hand, GY ranged from 253 kg ha^−1^ to 3070 kg ha^−1^ under NS condition (Table 1). Comparing with the mean GY of the NS trial in the 2012 DS (2156 kg ha^−1^), 70% and 69% GY reduction was noted in 2012 DS and 2013 DS RS trials, respectively (Table 2). Transgressive segregants for GY under RS conditions were also observed (Table 3).

**Table 1:** Descriptive trait statistics for parents (Kali Aus, IR64, and MTU1010) and mapping populations subjected to stress and non-stress conditions

** Population**	**Trait**		**2012 DS RS**	**2013 DS RS**	**2012 DS NS**	**2013 DS NS**
KaliAus/2*MTU1010	GY	Kali Aus	874	727	2658	2178
		MTU1010	465	419	3119	2809
		Population mean	664	638	2156	1947
		Highest line	1654	2650	3385	3973
		Lowest line	118	159	166	442
		LSD_0.05_	472	381	1193	1402
	PH	Kali Aus	74	55	91	88
		MTU1010	80	62	89	85
		Population mean	85	61	93	90
		Highest line	111	82	128	113
		Lowest line	62	43	70	58
		LSD_0.05_	23	13.4	22	20
	DTF	Kali Aus	84	85	74	79
		MTU1010	92	95	78	81
		Population mean	86	102	77	82
		Highest line	110	121	88	91
		Lowest line	77	82	69	74
		LSD_0.05_	9	15.6	5	8
Kali Aus/2*IR64	GY	Kali Aus	910	759	2731	2201
		IR64	495	335	1432	2874
		Population mean	722	582	1454	1604
		Highest line	1890	2063	3070	4168
		Lowest line	123	188	253	284
		LSD_0.05_	557	312	1117	1229
	PH	Kali Aus	68	101	90	87
		IR64	61	85	84	91
		Population mean	76	100	107	90
		Highest line	106	87	122	111
		Lowest line	50	36	73	62
		LSD_0.05_	22	9	24	21
	DTF	Kali Aus	87	85	76	80
		IR64	92	93	80	88
		Population mean	79	67	79	84
		Highest line	104	116	95	102
		Lowest line	80	75	68	75
		LSD_0.05_	11.2	15.9	8	9

2012 DS RS: 2012 dry season reproductive stress, 2013 DS RS: 2013 dry season reproductive stress, 2012 DS NS: 2012 dry season non-stress, 2013 DS NS: 2013 dry season non-stress, GY: grain yield (Kg h^-1^), PH: plant height (cm), DTF: days to flowering (d).

**Table 2:** Comparison of overall mean values of grain yield, plant height, and days to flowering under stress (2012 DS and 2013 DS) and non-stress (2012 DS) conditions

**Water condition**	**Grain yield (kg ha**^**−1**^**)**	**RYR (%)**	**Plant height (cm)**	**HR (cm)**	**Days to flowering**	**FD**
Kali Aus/2*MTU1010						
2012 DS NS	2156	-	93	-	77	-
2013 DS RS	638	70	61	34	102	25
2012 DS RS	664	69	85	9	86	9
Kali Aus/2*IR64						
2012 DS NS	1454	-	107	-	79	-
2013 DS RS	582	60	100	7	67	−12
2012 DS RS	722	50	76	29	79	0

2012 DS NS: 2012 dry season non-stress, 2012 DS RS: 2012 dry season reproductive stress, 2013 DS RS: 2013 dry season reproductive stress, RYR: relative yield reduction, HR: height reduction, FD: days of flowering delayed.

**Table 3:** Values of mean yield, mean DTF, and mean PH of selected promising genotypes for the two populations

**Population**	**Year**	**MTU1010**	Kali Aus/2*MTU1010	**IR64**	Kali Aus/2*IR64
**Promising lines selected (no.)**			**10**		**10**
GY (kg ha^−1^)	2012 DS RS	465	925	495	947
	2013 DS RS	419	1084	335	1023
	2012 DS NS	3119	3230	1432	2315
	2013 DS NS	2733	3009	2178	2418
Best line	2012 DS RS	465	1654	495	1890
	2013 DS RS	419	2650	355	2063
	2012 DS NS	3115	3385	1432	3070
	2013 DS NS	2733	4237	2178	3268
PH	2012 DS RS	80	90	61	78
	2013 DS RS	62	68	85	87
	2012 DS NS	89	101	84	90
	2013 DS NS	92	96	90	95
Best line	2012 DS RS	80	109	61	92
	2013 DS RS	62	78	85	86
	2012 DS NS	89	112	84	110
	2013 DS NS	92	107	90	103
DTF	2012 DS RS	92	83	92	88
	2013 DS RS	95	94	93	86
	2012 DS NS	78	75	80	77
	2013 DS NS	82	80	85	83
Best line	2012 DS RS	92	79	92	82
	2013 DS RS	95	91	93	78
	2012 DS NS	78	69	80	67
	2013 DS NS	82	72	85	74

2012 DS RS: 2012 dry season reproductive stress, 2013 DS RS: 2013 dry season reproductive stress, 2012 DS NS: 2012 dry season non-stress, 2013 DS NS: 2013 dry season non-stress,GY: grain yield (Kg h^−1^), PH: plant height (cm), DTF: days to flowering (d).

Best line – line combining high yield potential and good yield under drought.

The response of genotypes in terms of agronomic parameters differed significantly under both RS and NS conditions. The mean PH of Kali Aus/2*MTU1010 mapping population was 85 cm in 2012 DS; in 2013, the mean PH was 61 cm under RS and 93 cm under NS conditions, respectively (Table 1). For the Kali Aus/2*IR64 mapping population, the recorded mean PH was 76, 100, and 107 cm during 2012 DS, 2013 DS under RS, and 2012 NS, respectively. DTF was significantly affected by drought stress as reflected by a flowering delay of as many as 22 and 18 days (2012 DS) and 28 and 21 days (2013 DS) for Kali Aus/2*MTU1010 and Kali Aus/2*IR64 populations, respectively.

### Correlation and broad-sense heritability of traits under drought-stress condition

For the Kali Aus/2*MTU1010 population, GY was negatively correlated to DTF in both RS years (2012 DS and 2013 DS) and NS condition (Table 4). Similarly, a negative correlation between GY and DTF in all treatments for the Kali Aus/2*IR64 population was observed (Table 4). PH was positively correlated to GY in both RS and NS conditions in the Kali Aus/2*IR64 population and Kali Aus/2*MTU1010 population (Table 4).

**Table 4:** Correlation analysis between grain yield and agronomic traits of mapping populations derived from Kali Aus/2*IR64 under drought stress and non-stress conditions

**Population**		**DTF**			**PH**		
		**2012 DS RS**	**2013 DS RS**	**2012 DS NS**	**2012 DS RS**	**2013 DS RS**	**2012 DS NS**
Kali Aus/2*MTU1010	GY	−0.40**	−0.25*	0.59**	0.37*	0.68**	0.51**
Kali Aus/2*IR64	GY	−0.50**	−0.37*	−0.22	0.16	0.54**	0.62**

*Significance at 5%; **significance at 1%.

2012 DS RS: 2012 dry season reproductive stress, 2013 DS RS: 2013 dry season reproductive stress, 2012 DS NS: 2012 dry season non-stress, GY: grain yield (Kg h^−1^), PH: plant height (cm), DTF: days to flowering (d).

The combined heritability of two years for GY for Kali Aus/2*MTU1010 population was moderate, 0.51 and 0.56 during RS and NS conditions, respectively. For the Kali Aus/2*IR64 population, combined *H* for GY under drought stress was 0.48 and 0.58 during RS and NS conditions, respectively.

### Bulk segregant analysis

**Figure 1:**
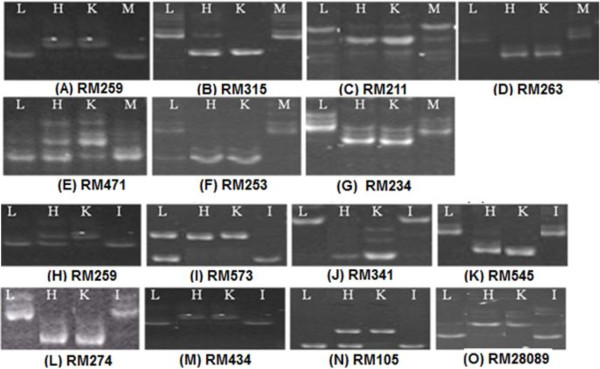
Simple Sequence Repeat (SSR) markers showing significant banding patterns between tail bulks (High – H, and Low – L), parents (Kali Aus – K, MTU1010 – M), (A-G) and (K - Kali Aus/donor parent; I – IR64/sensitive parent) (H-O).

In the Kali Aus/2*MTU1010 mapping population, seven out of the 109 markers used were found significant in BSA. These markers were RM259 (29.18 cM) and RM315 (143.9 cM) on chromosome 1, RM211 (7.92 cM) and RM263 (101.4 cM) on chromosome 2, RM471 (73.8 cM) on chromosome 4, RM253 (21.27 cM) on chromosome 6, and RM234 (99.9 cM) on chromosome 7. These markers showed high-bulk bands and low-bulk bands similar to those of Kali Aus (tolerant, donor) and MTU1010 (sensitive, recipient), respectively (Figure 1). In the Kali Aus/2*IR64 mapping population, eight markers showed differential banding patterns in high and low bulks, which corresponded to those of the tolerant (Kali Aus) and sensitive (IR64) parents (Figure 1). The markers identified were RM259 on chromosome 1, RM573 and RM341 on chromosome 2, RM545 on chromosome 3, RM274 on chromosome 5, RM434 and RM105 on chromosome 9, and RM28089 on chromosome 12.

### QTL analysis

#### GY QTL in the Kali Aus/2*MTU1010 population

**Figure 2:**
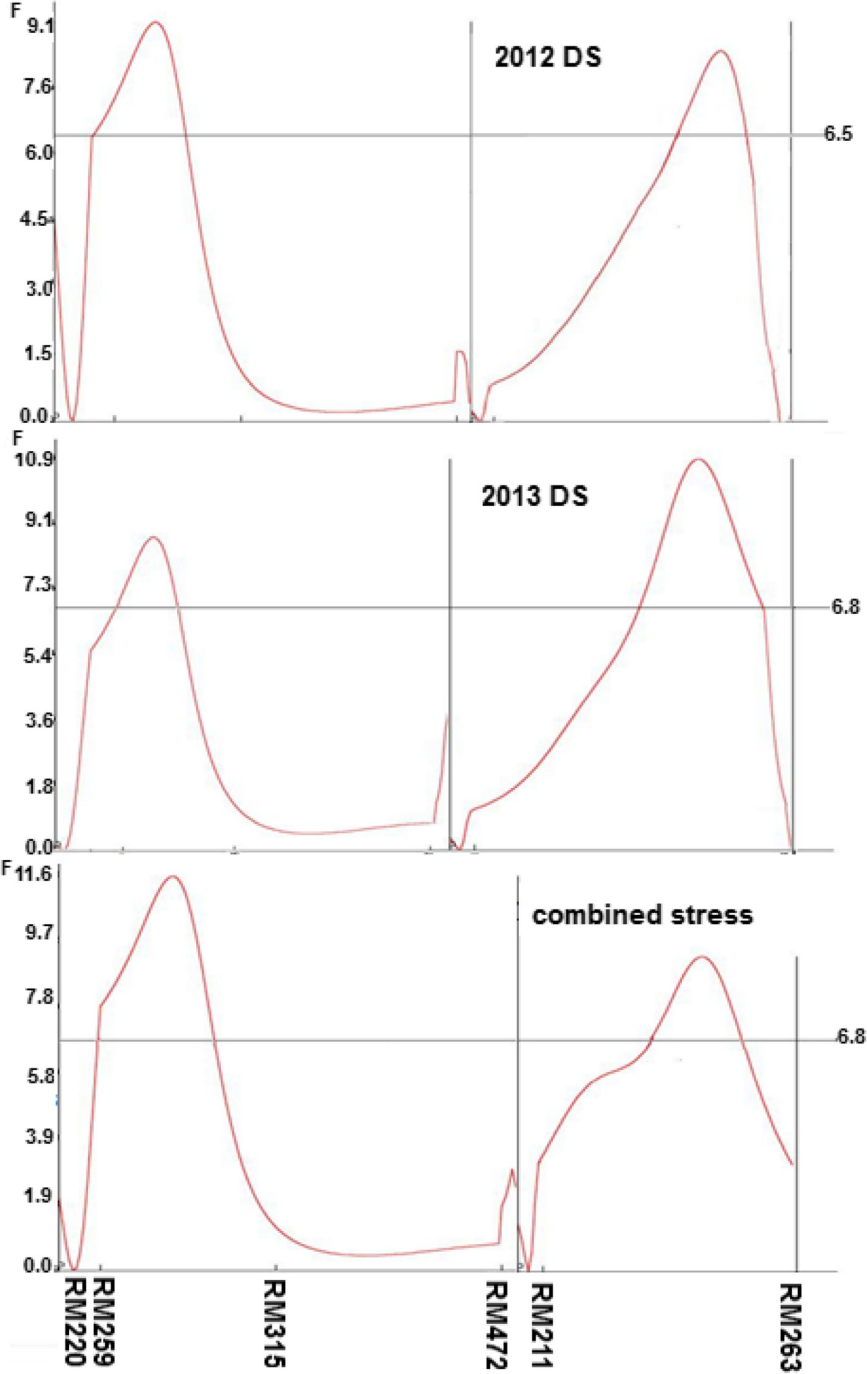
***F-*static curve indicating consistent QTL for grain yield (*qDTY***_***1.2 ***_**and *qDTY***_***2.2***_) of Kali Aus/2*MTU1010 mapping population under stress 2012, stress 2013, and combined stress conditions located on chromosome 1 and 2. Genetic distance (cM) between two markers is exhibited on X-axis while horizontal line corresponds for critical *F*-value (P < 0.01).

QTL with consistent effect (*qDTY*_*1.2*_ and *qDTY*_*2.2*_) for GY were mapped using CIM on chromosomes 1 and 2. These QTL are positioned at 50.2 and 72.9 cM flanked by markers RM259–RM315 and RM211–RM263, respectively (Figure 2, Table 5). The additive effect of *qDTY*_*1.2*_ on grain yield represented 17 and 19% of the trial mean for 2012 DS and 2013 DS, respectively. The *qDTY*_*2.2*_ exerted a positive additive effect of 21 and 30% of the trial mean for 2012 DS and 2013 DS, respectively under RS. The combined analysis showed a higher additive effect (19 and 24% of the trial mean) compared with both RS years, indicating a valid consistency for effect of QTL on GY under RS. The increase in GY for RS can be attributed to the Kali Aus allele, the tolerant parent, as reflected by the GY improvement of lines with QTL in contrast to lines without the QTL (Table 6). The consistent effect of QTL for GY under the respective RS years and in combined RS over 2 years is supported by the significant F static values (P > 0.01). At *qDTY*_*1.2*_, Kali Aus homozygotes significantly outyielded the MTU1010 homozygotes under RS condition for RM315 and RM259, individually and combined, with 22, 15, and 32%, respectively, in 2012 DS and 15, 12, and 33% GY advantage in 2013 DS (Table 6). Similarly, Kali Aus homozygotes for *qDTY*_*2.2*_ significantly outyielded the MTU1010 homozygotes under RS condition for RM211 and RM263, individually and combined, with 14, 9, and 20%, respectively, in 2012 DS and 13, 15, and 20% GY advantage in 2013 DS. *qDTY*_*1.2*_ and *qDTY*_*2.2*_, combined, exhibited a GY improvement of 26 and 33% in 2012 DS and 2013 DS, respectively (Table 6).

**Table 5:** QTL identified under upland drought-stress conditions in two populations

**Trait**	**QTL**	**Year**	**Flanking markers**	**Position (cM)**	**Additive effect (%)**	**F value**	**R**^**2 **^**(%)**
Kali Aus/2*MTU1010							
GY	*qDTY*_*1.2*_	2012 DS RS	*RM259*–RM315	50.2	17	9.0	6.3%
		2013 DS RS		50.2	19	8.2	4.5%
		Combined stress		50.2	19	11.6	7.1%
	*qDTY*_*2.2*_	2012 DS RS	*RM211*–RM263	72.2	21	9.3	6.2%
		2013 DS RS		72.9	30	10.9	6.0%
		Combined stress		72.9	24	8.6	5.8%
DTF	*qDTF*_*2.2*_	2012 DS RS	*RM211*–RM263	72.9	−3.8	8.4	4.5%
		2013 DS RS		73.9	−4.0	7.7	4.0%
		Combined stress		72.9	−3.7	12.3	7.8%
PH	*qPH*_*1.1*_	2012 DS RS	RM315–*RM431*	144.0	2.0	7.5	4.6%
		2013 DS RS		145.0	4.2	16.8	8.3%
		Combined stress		144.0	2.5	17.1	9.1%
Kali Aus/2*IR64							
GY	*qDTY*_*1.3*_	2012 DS RS	RM488–*RM315*	109.2	14	9.0	6.5%
		2013 DS RS		108.2	18	7.0	7.7%
		Combined stress		110.2	15	8.9	5.3%
	*qDTY*_*2.3*_	2012 DS RS	RM263–*RM573*	106.2	10	7.7	5.0%
		2013 DS RS		107.4	11	5.2	4.8%
		Combined stress		106.4	11	9.7	7.4%
		2012 DS NS	RM263–*RM573*	108.4	8.3	8.0	4.3%
		2013 DS NS		108.6	12	5.4	4.0%
		Combined non-stress		108.8	7	7.8	6.3%

2012 DS RS: 2012 dry season reproductive stress, 2013 DS RS: 2013 dry season reproductive stress, 2012 DS NS: 2012 dry season non-stress, 2013 DS NS: 2013 dry season non-stress, DTF: days to flowering; PH: plant height; GY: grain yield; additive effect depicted as % of mean trial. The marker in Italics font style the markers closet to peak.

**Table 6:** Yield improvement of genotypes possessing QTL (QTL +) for grain yield under reproductive stress over lines not possessing QTL (QTL -) for the two populations

**QTL**	**Trial**	**Markers**	**QTL (+)**	**QTL (−)**	**MTU1010/IR64**	**Yield improvement (%)**
Kali Aus/2*MTU1010						
*qDTY*_*1.2*_	2012 DS RS	RM315	568	441	465	22
		RM259	535	458	465	15
		RM259 + RM315	614	348	465	32
	2013 DS RS	RM315	483	406	419	15
		RM259	468	438	419	12
		RM259 + RM315	555	392	419	33
*qDTY*_*2.2*_	2012 DS RS	RM211	528	428	465	14
		RM263	505	440	465	9
		RM211 + RM263	559	351	465	20
	2013 DS RS	RM211	473	451	419	13
		RM263	482	446	419	15
		RM211 + RM263	504	419	419	20
*qDTY*_*1.2*_	2012 DS RS	RM259 + RM315	586	350	465	26
_*+*_		+				
*qDTY*_*2.2*_		RM211 + RM263				
*qDTY*_*1.2*_	2013 DS RS	RM259 + RM315	557	406	419	33
_*+*_		+				
*qDTY*_*2.2*_		RM211 + RM263				
Kali Aus/2*IR64						
*qDTY*_*1.3*_	2012 DS RS	RM315	576	377	495	16
		RM488	554	418	495	12
		RM488 + RM315	590	332	495	19
	2013 DS RS	RM315	395	350	335	18
		RM488	409	343	335	22
		RM488 + RM315	426	366	335	27
*qDTY*_*2.3*_	2012 DS RS	RM573	570	418	495	15
		RM263	526	440	495	6
		RM573 + RM263	586	320	495	18
	2013 DS RS	RM573	418	362	335	24
		RM263	429	357	335	28
		RM573 + RM263	452	322	335	35
*qDTY*_*1.3*_	2012 DS RS	RM488 + RM315	588	326	495	19
_*+*_*qDTY*_*2.3*_		+				
		RM573 + RM263				
*qDTY*_*1.3*_	2013 DS RS	RM488 + RM315	439	344	335	31
_*+*_*qDTY*_*2.3*_		+				
		RM573 + RM263				

2012 DS RS: 2012 dry season reproductive stress, 2013 DS RS: 2013 dry season reproductive stress, 2012 DS NS: 2012 dry season non-stress, 2013 DS NS: 2013 dry season non-stress.

QTL (+): genotypes possessing QTL donor allele for grain yield under reproductive stress.

QTL (−): genotypes not possessing QTL donor allele for grain yield under reproductive stress.

#### GY QTL in the Kali Aus/2*IR64 mapping population

**Figure 3:**
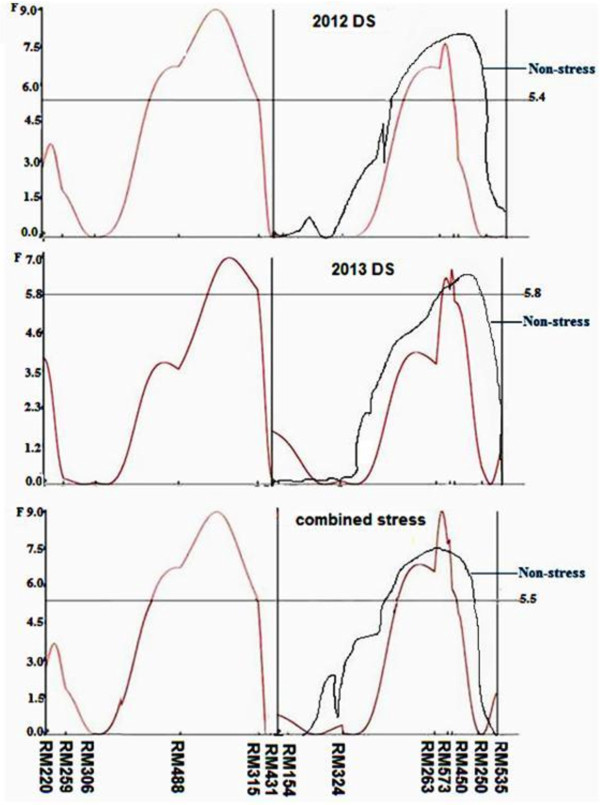
***F-*static curve indicating consistent QTL for grain yield (*qDTY***_***1.3***_**and *qDTY***_***2.3***_) of Kali Aus/2*IR64 mapping population under stress 2012, stress 2013, and combined stress conditions (Red line) and under non-stress 2012, non-stress 2013, and combined non-stress conditions (Black line) located on chromosome 1 and 2. Genetic distance (cM) between two markers is exhibited on X-axis while horizontal line corresponds for critical *F*-value (P < 0.01).

Significant and consistent-effect QTL *qDTY*_*1.3*_ and *qDTY*_*2.3*_ for GY were identified at chromosomes 1 and 2 flanked by markers RM488–RM315 and RM263–RM573 positioned at 109.4 cM and 104.4 cM (Table 5, Figure 3) with additive effect of 14 and 10% of trial mean under RS condition in 2012 DS, respectively; 18 and 11% under RS condition, respectively, in 2013 DS; and 15 and 11% of trial mean in combined stress, respectively. *qDTY*_*1.3*_ contributed 16, 12, and 19% increase in GY under 2012 DS RS condition; 18, 22, and 27% under 2013 DS RS condition, for RM315 and RM488, individually and combined, respectively, which can be attributed to the Kali Aus allele, the drought-tolerant parent (Table 6). For *qDTY*_*2.3*_, the homozygotes for RM573 and RM263 exhibited a GY improvement of 15, 6, and 18% for RS condition of 2012 DS and 24, 28, and 35% under 2013 DS RS condition, individually and combined, respectively. *qDTY*_*1.3*_ and *qDTY*_*2.3*_, combined, exhibited a GY improvement of 19 and 31% in 2012 DS and 2013 DS, respectively (Table 6).

#### QTL for DTF and PH

A significant QTL, *qDTF*_*2.2*,_ for DTF was mapped in Kali Aus/2*MTU1010 mapping population flanked by markers RM211–RM263 positioned at 67.9 cM in 2012 DS, 2013 DS and combined NS and at 73.9 cM in 2012 DS, 2013 DS and combined stress. The QTL for DTF co-located with *qDTY*_*2.2*_.

A QTL for PH was mapped in Kali Aus/2*MTU1010 under both environments in both seasons. It is located near marker RM315 and flanked by markers RM315–RM431 on chromosome 1 (Table 5).

#### Epistatic QTL

In addition to the major-effect, single-locus QTL (*qDTY*_*1.3*_ and *qDTY*_*2.3*_) for GY in the Kali Aus/2*IR64 mapping population, two major epistatic QTL for GY showing interaction between genomic locations on chromosome 1 at marker interval RM488–RM315 (*qDTY*_*1.3*_) and chromosome 2 at RM324–RM263 (*qDTY*_*2.1*_; identified previously by Venuprasad et al. [23]) in 2012 DS and 2013 DS RS with an additive effect of 35 and 46% of the population mean were identified. Four epistatic QTL for DTF showing interaction between genomic locations on chromosome 2 at marker intervals RM154–RM324 (*qDTF*_*2.1*_) (co-located with *qDTY*_*2.1*_; identified previously by Venuprasad et al. [23]) and RM263–RM573 (*qDTF*_*2.3*_) positioned at 4.3 cM and 101.4 cM, respectively, in 2012 DS, 2013 DS RS and on chromosome 1 at marker intervals RM259–RM306 (*qDTF*_*1.2*_) and on chromosome 2 at marker intervals RM450–RM250 (*qDTF*_*2.3*_) positioned at 32.2 cM and 128.2 cM, respectively, in 2012 DS, and 2013 DS NS were identified (Table 7).

**Table 7:** QTL interactions detected for grain yield and yield-contributing traits during 2 years of screening under upland conditions in Kali Aus/2*IR64 population

**Trait**	**Year**	**QTL**_**i**_	**Interval**_**i**_	**Position**_**i **_**(cM)**	**QTL**_**j**_	**Interval**_**j**_	**Position**_**j **_**(cM)**	**AA (%)**	**F**_**i**_**, F**_**j **_**value**	**P value**	**R**^**2 **^**(%)**
GY	2012 DS RS	*qDTY*_*1.3*_	RM488–RM315	108.2	*qDTY*_*2.1*_	RM324–RM263	53.6	35	3.8, 4.4	0.00017	6.6%
	2013 DS RS	*qDTY*_*1.3*_	RM488–RM315	108.2	*qDTY*_*2.1*_	RM324–RM263	60.6	46	4.0, 4.8	0.00009	6.2%
DTF	2012 DS RS	*qDTF*_*2.1*_	RM154–RM324	24.3	*qDTF*_*2.3*_	RM263–RM573	101.4	1.28	5.7, 4.3	0.00004	6.7%
	2013 DS RS	*qDTF*_*2.1*_	RM154–RM324	14.3	*qDTF*_*2.3*_	RM263–RM573	101.4	2.64	4.4, 3.9	0.00063	5.4%
	2012 DS NS	*qDTF*_*1.2*_	RM259–RM306	32.2	*qDTF*_*2.3*_	RM450–RM250	128.2	2.02	4.4, 3.3	0.000005	7.4%
	2013 DS NS	*qDTF*_*1.2*_	RM259–RM306	30.2	*qDTF*_*2.3*_	RM450–RM250	124.4	2.21	4.0, 5.7	0.00003	6.5%

2012 DS RS: 2012 dry season reproductive stress, 2013 DS RS: 2013 dry season reproductive stress, 2012 DS NS: 2012 dry season non-stress, 2013 DS NS: 2013 dry season non-stress.

QTL_i_ and QTL_j_: testing points i and j, respectively.

Interval_i_ and Interval_j_: the intervals of testing points i and j.

AA: additive-by-additive.

## Discussion

In both populations and years, yields were lower under reproductive stage drought stress compared to non-stress indicating very high stress levels. Such high stress levels were desirable because a high percentage reduction of yield is necessary to remove the effect of yield potential and clearly identify lines that are drought-resistant [9,21].

Bulk segregant analysis was able to identify significant markers linked to loci that exert an effect on GY under RS. Through the subsequent whole-population genotyping, the presence of major and consistent-effect QTL was confirmed. This confirms the finding that BSA is an effective approach in mapping QTL associated with rice GY under RS condition [23,24]. BSA is indeed a cost-effective scheme of identifying QTL alleles as considerable savings in time and inputs can be achieved in genotyping efforts, thus allowing resources to be allocated to more precise identification of QTL with large effects [35].

QTL from tolerant donors identified to show high effect against non-elite drought-susceptible genetic backgrounds are less likely to show high impact against high-yielding, elite genetic backgrounds [36]. In the case of drought, QTL × genetic background interaction has been reported to be a major factor limiting the use of QTL for MAB in rice [21]. In our study, the QTL (*qDTY*_*1.2*,_*qDTY*_*1.3*_, *qDTY*_*2.2*,_ and *qDTY*_*2.3*_) were identified using one common donor parent (Kali Aus) in the backgrounds of mega varieties IR64 and MTU1010. The QTL *qDTY*_*1.2*_ and *qDTY*_*1.3*_ was found to be located near to the earlier reported QTL *qDTY*_*1.1*_[24]. It is noteworthy that *qDTY*_*1.1*_ was reported previously to have significant effect on GY under both RS and NS conditions in N22/Swarna, N22/IR64, and N22/MTU1010 backgrounds in two seasons [24]. QTL for GY under RS has also been reported by Kumar et al. [22] and Ghimire et al. [25] in this region. *qPH*_*1.1*_ also showed a significant effect on PH under both environments in Kali Aus/2*MTU1010 populations in both seasons in our study similar to the study of Vikram et al. [24]. The allele that increased GY under RS was contributed by a comparatively susceptible parent in a study conducted by Kumar et al. [22]. However, the positive allele in our study was contributed by the tolerant parent, Kali Aus, similar to what Vikram et al. [24] and Ghimire et al. [25] found in their studies. Apart from this, the region has shown a rich diversity of QTL related to various drought-tolerance traits such as root-related traits [37], PH [21], root dry weight [38], grains per panicle [39], relative water content under drought [40], biomass, basal root thickness, and osmotic adjustment [41].

Two different QTL, *qDTY*_*2.2*_ and *qDTY*_*2.3*_, were mapped in Kali Aus/2*MTU1010 and Kali Aus/2*IR64, respectively, despite a common donor parent being used to develop both populations. The *qDTY*_*2.2*_ exerted a positive additive effect around 21 and 30% of the trial mean for 2012 DS and 2013 DS, respectively, under RS and *qDTY*_*2.3*_ showed additive effect of 10 and 11% of the trial mean for 2012 DS and 2013 DS, respectively. Combined analysis showed an additive effect of 24 and 11% of the trial mean for *qDTY*_*2.2*_ and *qDTY*_*2.3*_, respectively, indicating a valid consistency with respect to the effect of QTL on GY under RS. Several QTL have been previously reported to have a significant effect on GY under RS, which proved to be highly specific to the background parent [42]. Nonetheless, it is still possible that the effect of these QTL on GY could be validated in other genetic backgrounds. The mapped QTL can be introgressed into the background of drought-susceptible rice varieties to improve their drought tolerance through MAB. In the present study, the effect of *qDTY*_*2.3*_ was also observed on DTF under NS in addition to its effect on GY under RS*.* It is interesting to note that *qDTY*_*2.3*_ also increased GY significantly under severe RS in the N22/IR64 population [24]. Similarly, *qDTY*_*2.2*_ has earlier been identified to be contributed by Aday Sel, another traditional drought-tolerant donor from India, and has shown a high effect in the IR64 background. In previous studies, *qDTY*_*2.2*,_*qDTY*_*4.1*_*, qDTY*_*9.1*_*,* and *qDTY*_*10.1*_ contributed by donor Aday Sel have shown an effect in the IR64 background, [43]. In the present study, *qDTY*_*2.2*_ showed effect in the MTU1010 background and not in IR64 background, indicating the importance of interaction between QTL allele and genetic background in determining the effect of a QTL region. The study further indicates that, before imparting a marker-assisted introgression program for a complex trait such as GY under drought, it is necessary that individual QTL/QTL combinations with high effect on yield be identified or the effect of the identified QTL be validated. This can be efficiently achieved through the use of backcross derived line (BIL) populations as used in the present study, which shall enable breeders to identify the best QTL/QTL combinations effective against a particular background in the early backcross generations. The availability of such information enables easy identification of lines with QTL showing high yield under both irrigated and drought stress conditions as well as lines with QTL and high background recovery in the subsequent backcross generations.

*qDTY*_*2.3*_ showed 12 and 18% yield improvement under NS condition also in 2012 DS and 2013 DS, respectively. The effect of *qDTY*_*2.3*_ under both RS and NS makes it an important candidate for use in MAB to increase yield under both RS and NS conditions. Bernier et al. [44] reported the stability of *qDTY*_*12.1*_ across different environments; *qDTY*_*2.2*_ and *qDTY*_*4.1*_ together have been reported to contribute to increase GY under both RS and NS conditions [43]. In both cases, the increase in GY was high under RS and low under NS, which was also observed in the present study. The present and earlier findings clearly refute the earlier notion that introduction of drought tolerance leads to a reduction in yield potential. In fact, the precise marker-assisted introgression of QTL such as *qDTY*_*12.1*_, *qDTY*_*2.3*_ provides opportunities to improve yield under RS as well as under NS.

A significant QTL for DTF, *qDTF*_*2.2*_, was mapped in the Kali Aus/2*MTU1010 population under NS and RS, both flanked by markers RM211 and RM263, which co-located with *qDTY*_*2.2.*_ Similarly, a QTL for PH was mapped in the Kali Aus/2*MTU1010 under both NS and RS in both seasons and it co-located with earlier reported *qDTY*_*1.1*_[24] on chromosome 1. The co-location of QTL for GY/DTF and PH trait may be attributed to the effect of pleiotropy or very close linkage of genes controlling these traits. It may be possible that the variation caused by QTL governing DTF and/or PH located in these regions, in conjunction with the timing of stress imposed, affected grain yield. The co-location of grain yield and DTF/PH QTL provides a unique opportunity to use them in the background of traditionally favored semi-dwarf varieties with long duration to flowering to develop novel varieties adapted to specific environments. Coexisting chromosomal regions/loci governing different traits provide a unique opportunity for breeders to introgress such regions together as a unit into high-yielding lowland varieties through MAS/MAB and to develop cultivars possessing increased tolerance for drought conditions.

The lines with *qDTY*_*1.2*_ + *qDTY*_*2.2*_ and *qDTY*_*1.3*_ + *qDTY*_*2.3*_ QTL showed a mean yield advantage of 26–33 and 19–31% over lines without QTL in the Kali Aus/2*MTU1010 and Kali Aus/2*IR64 populations, respectively (Table 6). However, for breeding, the yield advantage of promising lines combining high yield potential and good yield under drought over that of recipient high-yielding parents MTU1010 and IR64 is important. The means of 10 promising lines from both Kali Aus/2*MTU1010 and Kali Aus/2*IR64 populations showed a yield improvement of 0.5 t ha^−1^ under drought over recipient parents MTU1010 and IR64 with at least a similar yield under NS condition, clearly showing that lines with QTL have a yield advantage over recipient parents under drought with no yield penalty under NS condition (Table 3). Further, the best line from each population showed a yield advantage of at least 1.1 and 1.4 t ha^−1^ under drought in Kali Aus/2*MTU1010 and Kali Aus/2*IR64 populations, respectively, with some yield advantage under NS condition also.

Genome-wide epistasis was identified in rice with the advent of molecular markers; in fact, an increasing number of results have revealed the importance of epistasis in yield traits [14,15,45,46]. Interestingly, in the present study, an epistatic interaction was observed for QTL for DTF flanked by markers RM154–RM324 and RM263–RM573 and was found to be co-located with *qDTY*_*2.1*_ (identified previously by Venuprasad et al. [23]) and *qDTY*_*2.3,*_ respectively in 2012 DS and, 2013 DS RS and other epistatic interaction between RM259–RM306 and RM263–RM250 in 2012 DS and 2013 DS NS. Similarly, two major epistatic QTL were identified for GY on chromosome 1 at marker interval RM488–RM315 (*qDTY*_*1.3*_) and on chromosome 2 at RM324–RM263 (*qDTY*_*2.1*_) in 2012 DS and 2013 DS RS. *qDTY*_*2.1*_, which was identified in a population derived from the cross Apo/2*Swarna [23], has previously been reported to affect yield under severe lowland drought. These results specify the role of the interaction of QTL with a large effect on GY with other loci with a small effect on GY/yield-contributing traits to further enhance GY under drought in upland ecosystems (Table 8). The possible interaction of major-effect QTL with other QTL as well as genetic background as revealed in the present study could be one of the possible reasons for the variable effect of QTL in different genetic backgrounds. The identification of such interactions may provide suitable answers to the environment/background-specific response of many of the identified QTL. Detection and pyramiding of two to three such interacting QTL with a large effect on grain yield under drought may provide wider adaptability of these QTL across genetic backgrounds and environments and could reduce to a certain extent the large QTL x environment and QTL x genetic background interactions often observed for drought.

**Table 8:** Effect of QTL classes on grain yield and DTF under upland conditions over 2 years of screening in Kali Aus/2*IR64 population

**QTL**	**Trial**	**QTL (+)**	**QTL (−)**	**IR64**	**Yield improvement (%)**
*qDTY*_*1.3*_	2012 DS RS	576	377	495	16
	2013 DS RS	395	350	335	18
*qDTY*_*2.1*_	2012 DS RS	526	434	495	06
	2013 DS RS	381	337	335	14
*qDTY*_*1.3*_ *+ qDTY*_*2.1*_	2012 DS RS	591	430	495	19
	2013 DS RS	406	346	335	21
*qDTY*_*1.3*_*+ qDTY*_*2.1*_ *+ qDTY*_*2.3*_	2012 DS RS	607	315	495	22
	2013 DS RS	419	308	335	25

2012 DS RS: 2012 dry season reproductive stress, 2013 DS RS: 2013 dry season reproductive stress.

QTL (+): genotypes possessing QTL donor allele for grain yield under reproductive stress.

QTL (−): genotypes not possessing QTL donor allele for grain yield under reproductive stress.

## Conclusions

The study identified large and consistent-effect QTL, *qDTY*_*1.2*_, *qDTY*_*1.*3_, qDTY_2*.2*_, and *qDTY*_*2.3*_ on GY under reproductive-stage drought stress and two epistatic QTL for GY and four for DTF. The study identified promising lines with a yield advantage of 1.0 t ha^−1^ or more under drought, clearly indicating that these identified QTL for GY under drought can be used to improve the GY of mega varieties MTU1010 and IR64, over different degrees of severity of drought stress through MAB and provide farmers improved varieties with good yield under drought and with no yield loss under non-stress conditions. The co-location of grain yield and DTF/PH QTL provides a unique opportunity to use them in the background of traditionally long-duration and short height but highly favored varieties to develop high-yielding varieties with reduced duration and to increase plant height that are suitable for cultivation under the present scenario of reduced water availability. The observed epistatic interaction for GY and DTF provides a means to uncover novel genetic networks affecting these traits.

## Abbreviations

BSA: Bulk segregant analysis; cm: Centimeter; CTAB: Cetyltrimethyl ammonium bromide; d: Days; DNA: Deoxyribonucleic acid; DS: Dry season; GY: Grain yield; IRRI: International rice research institute; MAB: Marker-assisted breeding; MAS: Marker-assisted selection; NPK: Nitrogen, phosphorus, and potassium; NS: Non-stress; PH: Plant height; PAGE: Polyacrylamide gel electrophoresis; PCR: Polymerase chain reaction; RIL: Recombinant inbred line; RS: Reproductive stress.

## Competing interests

The authors declare that they have no competing interest.

## Authors’ contributions

NS was involved in conceptualizing the experiment, analysis, interpretation of data, and drafting the article; AS, SD, and RKJ contributed to the revision of the manuscript; AK was involved in the design of the experiment and in the critical revision of the manuscript. TSC and PCM were involved in designing the experiment layout, recording observations, and revising the manuscript. All authors approved the final version of the manuscript.

## Supplementary Material

Additional file 1: Figure S1 Tensiometer reading during dry seasons of 2012 and 2013 at IRRI Experimental Area. Click here for file
