# Supplementary figures and images for: Identification and mapping of stable QTL with main and epistasis effect on rice grain yield under upland drought stress
Source: BMC Genet. 2014 May 27;15:63. doi: 10.1186/1471-2156-15-63 (PMC4048250; doi:10.1186/1471-2156-15-63)

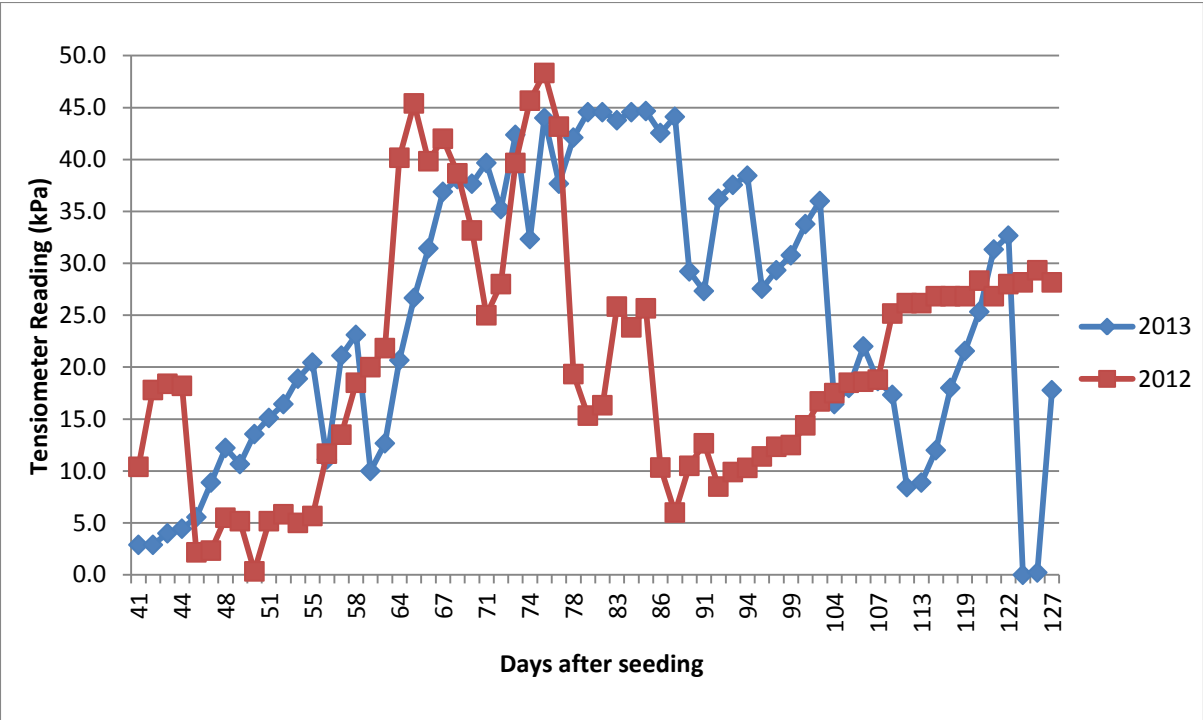

Supplementary Figure 1

Supplement: Additional file 1: Figure S1 — Tensiometer reading during dry seasons of 2012 and 2013 at IRRI Experimental Area. [file 1471-2156-15-63-S1.pdf]
